# IEEN workshop report: Teaching and learning in interdisciplinary and empirical ethics

**DOI:** 10.1177/1477750913499493

**Published:** 2013-09-10

**Authors:** Jonathan Ives, John Owens, Alan Cribb

**Affiliations:** 1 University of Birmingham, UK; 2 Centre for Public Policy Research, King’s College London, UK

**Keywords:** bioethics, education, interdisciplinarity, pedagogy, standards

## Abstract

Bioethics is an interdisciplinary field that accommodates a broad range of perspectives and disciplines. This inherent diversity sets a number of challenges for both teachers and students of bioethics, notably in respect to the appropriate aims and methods of bioethics education, standards and criteria for evaluating performance and disciplinary identity. The Interdisciplinary and Empirical Ethics Network (IEEN) was established, with funding from the Wellcome Trust, to facilitate critical and constructive discussion about the ongoing development of bioethics as an evolving field of interdisciplinary study. In November 2012 the IEEN organised a workshop at the University of Birmingham to discuss the issues relating to teaching and learning in interdisciplinary and empirical bioethics. This paper reports on that meeting.

Bioethics is an inherently diverse field that accommodates contributions from a broad range of disciplinary backgrounds and traditions.^1^ This diversity raises questions about the aims and methods of bioethical research, particularly since those carrying out this research may seek to ‘do bioethics’ in different ways and in the pursuit of a different set of aims. For example, whilst some may consider bioethics to be a site for normative reasoning and conceptual analysis, others may treat research in bioethics as a means of highlighting areas of ethical controversy and others may be more interested in using bioethics to look for ethical ‘solutions’ to real-world practical problems. The Interdisciplinary and Empirical Ethics Network (IEEN) was established, with funding from the Wellcome Trust, to facilitate critical and constructive discussion around the nature of this disciplinary diversity and shift focus away from the ‘empirical turn’, towards the ongoing development of bioethics as an evolving field of interdisciplinary study. Membership of the network is open, and it can be joined by e-mailing the authors of this paper. New members are added to the mailing list, and will be notified of future events and other items of interest relevant to the group’s primary activities. Further details of the network are available online at http://www.birmingham.ac.uk/research/activity/mds/projects/HaPS/PCCS/MESH/ieen/index.aspx

Increased attention to aims and methods in interdisciplinary and empirical ethics (IEE) has inevitably led to questions being asked about how best to prepare researchers to undertake this kind of research. These questions are complicated by the lack of consensus on aims and methods, and whilst the two issues cannot be divorced entirely, it is still possible to consider teaching and learning without having resolved the question about aims and methods. It may also be possible to use thinking about the former to shed light on the latter.

Accordingly, moving on from the first IEEN workshop in April 2012, which considered aims and methods in IEE, the second workshop considered questions about teaching and learning in this rich interdisciplinary field and reflected on the challenges, pitfalls and opportunities involved in training researchers to conduct ethics research in a genuinely interdisciplinary way. Speakers were invited to consider the following themes:

1. the nature of interdisciplinarity;
2. the significance of teaching and learning for disciplinary identity;
3. the possibility of training of researchers conversant and competent in multiple disciplinary traditions and
4. the pedagogy of interdisciplinary teaching and learning.

The day was structured into two sessions, each comprising three 20-minute talks and an hour for general discussion. The morning session included three talks by academics involved in teaching and supervising research in IEE, and the afternoon session included three talks by researchers and students, at different stages of their learning career, who reflected on the process of learning in an interdisciplinary ethics context.

Lucy Frith (University of Liverpool) began the day with a discussion of what a ‘discipline’ is. She outlined five possible criteria:

1. a common object of research (though this may be shared with others);
2. a body of specialised knowledge;
3. specific terminology;
4. specific research methods;
5. some institutional manifestation, e.g. departments/facilities/locations/points of situation,

and considered whether, using these criteria, IEE could be described as a ‘discipline’ in its own right. Lucy argued that it might not, and might better be characterised as a ‘field’ or a subset containing a number of different disciplines. This conclusion was then examined in light of an historical overview, which noted how ‘disciplines’ are in a state of flux, and their development, and re-development, is linked to institutional and political factors, and rarely driven by academic content alone. This historical analysis was followed by consideration of the ‘function’ of disciplines, which might include:

- disciplines as disciplining (Foucault);
- disciplines as exclusive and exclusionary and
- disciplines as territorial and a source of tribalism.

Citing Wenger,^2^ who conceptualised disciplines as ‘communities of practice’, Lucy then asked whether IEE could be seen as a discipline in this functional sense, given that the label does seem to have important role in helping to form a community of researchers who have certain kinds of aims and certain kinds of academic practices. Lucy argued that working within a ‘discipline’ of IEE might require familiarity with a broader canon of work and a wider community of practice than may be found in more narrowly defined areas, but with the right resources, recognition and encouragement this can be achieved.

Barbara Prainsack (King’s College London) picked up where Lucy left off, and reflected on why, in the context of a ‘discipline’ in which one may have to engage with a broader canon of knowledge and wider range of practices than would ordinarily be required, we would want to pursue IEE and train researchers accordingly.

Barbara focused particularly on one key question: Does interdisciplinarity produce ‘better’ knowledge than conventional disciplinary research? She noted that this is hard to assess empirically, and that conceptions of ‘better’ will be subject to interpretation. Notwithstanding this, she posited two scenarios, optimal and worst:

– An optimal scenario might see interdisciplinarity create better, more concrete forms of knowledge which might not be able to be captured by conventional forms of research.
– A worst case scenario, in which interdisciplinarity is used to discipline people. For example, people who are seen as falling short of disciplinary standards (e.g. because they publish in the ‘wrong’, interdisciplinary journals) may be pushed out to the margins to do interdisciplinary work.^3^

The situation is complicated even further by increasing numbers of academics moving towards a model of ‘transdisciplinarity’, in which collaboration is sought and practised with people outside of traditional academic circles. This goes beyond treating people outside of academia as mere sources of data and/or information, and instead views external figures as a potential source of collaboration in the process of carrying out research itself. In particular, Barbara referred to the success of the recent Foldit project (http://fold.it/portal/) to illustrate the potential value that ‘crowd-sourcing’ techniques might have for academic research. Barbara also argued that facilitating interdisciplinarity should not be limited to transposing concepts and methods out of the disciplinary contexts in which they are based and using them in other disciplines, which can lead to inappropriate and poor application of methods and practices.

Barbara concluded by considering what future an interdisciplinary and empirical bioethics might have, and painted a picture of interdisciplinarity in which Bioethics incorporates new sources of knowledge and expertise. She finished by posing an important, single question: *What kind of Bioethics do we want next*?

Barbara was followed by Richard Huxtable (Bristol University) and Jonathan Ives (University of Birmingham). They gave a joint presentation that suggested a potential answer to Barbara’s question by moving the discussion on to appropriate teaching practices in the inter/intra/trans disciplinary context of IEE. In turn, each outlined their respective institution’s intercalated degree in medical ethics, and described different approaches to achieving a blending of disciplinary perspectives, and the value of doing so.

Richard focused on reporting a pilot study, kindly funded by the Alumni Fund of the University of Bristol and conducted in collaboration with his colleagues Zuzana Deans and Jane Reece, which looks at the benefits of intercalation in Ethics and Law. The pilot study suggested that intercalaters in healthcare ethics and law value the experience in terms of the benefits it confers to themselves as individuals, as medical students and as doctors to be. These benefits manifested in the practical value of acquiring transferable skills in argument, presentation skills, independent working skills and also character-based benefits such as confidence, becoming more rounded and mature, and changing one’s outlook on life. The experience was therefore felt to be edifying both for one’s knowledge and skills, but also one’s character. The costs of intercalation were also discussed, and these centred around the financial cost of spending another year in higher education, and the stress of reintegrating into medical learning once the intercalation has ended. Richard then reflected on whether these benefits, reported by students, said something about bioethics itself – and whether training in bioethics engendered positive attitudes about dialogue, openness, respect and listening to others. This led Richard to ask important questions about how students ought to be trained at this level, to what extent skills or knowledge ought to be the focus, or whether the development of character ought to be an explicit aim. Do the benefits outlined stem from training in a single discipline, or is an interdisciplinary focus, with the necessary focus on dialogue, competing perspectives and compromise that comes with it, important? He finished by highlighting the difficulty of achieving this in a limited timeframe, and the challenge involved in assessment.

Picking up on this, Jonathan gave an overview of the aims, pedagogy and assessment process of the Birmingham programme. The first semester is devoted to three taught modules, with teaching and assessment that are aimed at preparing students to undertake their own independent research in the second semester. One of these modules, ‘an introduction to research methods in bioethics’, introduces skills in ethical, philosophical and legal reasoning, reading and writing, qualitative research methods and, importantly, approaches to interdisciplinary empirical ethics research. The latter comprises three 2-hour sessions, devoted to exploring various rationales for IEE, key meta-ethical and epistemology issues, methods and methodologies for integrating ethical analysis with empirical social science research, and practical issues in interdisciplinary ethics research. Many first semester assessments are designed to help students work up ideas for their dissertation project, which is then carried out in the second semester. Students can choose whether to do a library-based project, or to conduct research that involves a combination of theoretical and empirical work. Jon reflected on the fact that this programme is very demanding, and it requires students to work independently and creatively. The benefit of this is that as well as acquiring disciplinary skills and knowledge, students develop and practise a host of other transferable skills – the most significant of which, perhaps, is the ability to deal with uncertainty. It is possible, Jon said, to predict precisely when students will be stressed and worried, and over time the programme has instituted various additional teaching and pastoral seminar slots to deal with this anxiety. Once the programme is completed, student feedback is invariably positive, and very similar to that described by Richard.

During the discussion that followed, one key point to arise was that disciplines are important because they provide us with standards for evaluation. Mark Sheehan advanced this point, suggesting that interdisciplinary subjects have a potential problem if they do not incorporate those standards. The problem is that these standards may be in competition, and there is no clear way of determining which standards must take priority. Barbara Prainsack responded, noting that peer reviews often refer to notions of disciplinary standards. Given, however that there is not one set of dominant standards in bioethics – which is a feature of interdisciplinary – it becomes difficult to evaluate interdisciplinary work. Jonathan Ives suggested that we might need to think of standards as not relating to a specific discipline, but as related to the specific research task and the particular questions faced. In an interdisciplinary bioethics, the evaluation of the way in which specific questions are answered may need to be privileged over general disciplinary concerns, and the standards used to evaluate the work determined flexibly *post hoc*. Since providing adequate answers to the different types of questions within bioethics can demand the application of different sorts of methods and techniques, often to different specified standards, the development of the field of interdisciplinary and empirical bioethics may require a degree of flexibility, pragmatism and interdisciplinary knowledge from those involved.

The second session began with a talk from Rachel Davies and Sophie Taylor, both medical students at the University of Birmingham who intercalated in Ethics and Law. They described their experience of learning on the Birmingham programme and used an analogy to illustrate. Learning how to do research in an interdisciplinary bioethics, they argued, is like trying to bake a cake. Only, you have a list of ingredients but no clear instruction on how to combine them, nor what the final bake should look or taste like. They described trying to undertake research that conforms to the canon of bioethics literature, but highlighted the difficulty in doing this, given that the canon in IEE is ill-defined and ambiguous. They highlighted that inexperience makes engagement with the literature and methods difficult, and they found themselves, whilst trying to learn ‘how to do bioethics’, lost in a sea of competing disciplinary aims, methods, outcomes and ideas.

Rachel and Sophie then reflected on the various ways in which ‘doing’ bioethics might be taught, and concluded that problem-based learning (PBL) had significant benefits over traditional didactic approaches. PBL approaches, they suggested, encourage the kind of independence and experimentation that may be necessary for conducting this kind of interdisciplinary research, and are beneficial in the long term. They noted, however, that the process of learning in this way was daunting and challenging. Whilst they felt that the programme learning outcomes were well defined, in the absence of a clearly defined canon they still struggled to understand exactly what was expected of them.

Andrew Papanikitas (King’s College London) followed, with a presentation that described his experience of learning and doing postgraduate bioethics research on bioethics scholarship and moral development in general practice, in an interdisciplinary context. He described the phenomenon of ‘learner anxiety’, which tied in with the previous two presentations, and linked this to what he felt was a lack of clarity about what to foreground as the key subject matter in IEE education – theoretical ethics, method or empirical work? Andrew identified three key ‘learning anxieties’:

1. *Methodological anxiety*: the feeling of being adrift in a heterodoxical crowd of disciplines, and never being sure what criteria of rigour should be applied, or even what style to write in.
2. *Anxiety in relation to the field*: the tension one feels in navigating the roles of the ‘ambassador’ who has to represent participants’ agendas but not be exploited by them, the ‘spy’ who risks exploiting participants, and the ‘native’ who risks becoming so familiar with the field that he is unable to take a critical view.
3. *The ‘so what’ anxiety*: the difficulty in answering the question of relevance in relation to one’s work.

He noted that he empathised with the experience of Rachel and Sophie, and felt similarly adrift at times, characterising his experience as a learner in terms of trying to ascertain exactly what was expected of him. He suggested that fora such as the IEEN were important, and acted, to some extent, as an antidote to these anxieties because they facilitate the realisation that these anxieties are shared by others, and offer opportunities for supportive discussion and critique.

Last to speak was Kerry Woolfall (Liverpool University), who described her ongoing postdoctoral experience working on a number of interdisciplinary bioethics projects. Kerry recounted that she began this work with a background in social scientific research but having had no experience working in bioethics. She outlined the ‘CONNECT’ and ‘Recruit’ projects, which have the combined aims of integrating empirical and normative analysis to develop normative guidelines to help to optimise recruitment into paediatric emergency care trials. Her methodology is based on that described by Ives and Draper^4^ and involves a review of the bioethical literature with concurrent three-phase data collection (questionnaires, a trial and interviews) with a view to achieving some kind of synthesis of theoretical and practical concerns.

Kerry noted the lack of available training specifically in interdisciplinary bioethics research, and suggested that given the increasing number of people coming to work in this way there was a national need for such courses. She identified three particular areas where teaching and training was required:

1. normative and empirical synthesis/integration;
2. production of normative guidelines and outcomes from that synthesis/integration and
3. ethical issues in interdisciplinary research.

The discussion that followed picked up on this, considering the possibility of developing short training courses which could address some of the disciplinary, theoretical and methodological challenges facing both students and teachers of IEE. For example, a week-long course on empirical research methods may be of benefit to bioethicists from philosophical backgrounds seeking to develop capabilities in IEE. Likewise, a short course in conceptual analysis and normative reasoning may help bioethicists from social scientific backgrounds develop confidence in and familiarity with the philosophical aspects of IEE. The question of who is responsible for developing this training was raised, with various contenders discussed, ranging from the idea that teaching should be reactive to the learner (and therefore dictated by the learner), to the idea that the funders of interdisciplinary research should take some responsibility for developing and defining appropriate training packages.

## Summary

Alan Cribb brought the workshop to a close by summarising the major themes that emerged from the event. His remarks drew on the presentations of Rachel, Sophie, Andrew and Kerry and, amongst other things, highlighted the significance of anxiety in teaching and learning.

1. Learning is often associated with anxiety but, for a variety of reasons, teaching and learning in IEE is especially characterised by anxiety, even though it is often a rewarding and ultimately positive experience. In some respects traditional academic disciplines provide a form of security, because they can draw on their heritage, and well-established organisations and practices, to offer established sets of standards for planning and assessing teaching and learning.
2. By contrast, the multi-disciplinary nature of IEE can bestow teaching and learning with an inherent ambiguity and open-endedness which must first be acknowledged before it can be effectively negotiated in practice. On all sides, the development of teaching and learning in IEE requires awareness of the challenges involved which require students and teachers alike to develop the pragmatic, communicative and reflexive attitudes and attributes required to meet these challenges.

Overall, there was agreement that both teachers and learners, and possibly funders of research, have a responsibility to reflect upon the challenges of teaching and learning in IEE, and to develop effective pedagogic strategies. Whilst there may be good arguments for the creation of bespoke interdisciplinary training schemes, there are also arguments for keeping training separated along traditional disciplinary lines. The latter might ensure that, prior to undertaking interdisciplinary research, practitioners have sufficient understanding of the methods and standards of rigour of the ‘parent’ disciplines– something that some commentators have argued has been missing to date.^5^ This latter point, of course, might beg the question of how we think disciplines are, and should be, constructed, organised and ultimately evaluated.

These concluding themes were then used to introduce the agenda for the IEEN’s third workshop which focused on the nature of professionalism in bioethics, considering issues related to the following questions:

- What counts as doing the job of a bioethicist well?
- What are the qualities, skills and attributes required to be a good bioethicist and what is the relationship between expertise, skills standards and professionalism in bioethics?
- What is the public position of the bioethics profession? Could/ought bioethics be seen as analogous to other professional groups, especially in terms of some sort of underpinning institutional embodiment?
